# Fungal Communities on Standing Litter Are Structured by Moisture Type and Constrain Decomposition in a Hyper-Arid Grassland

**DOI:** 10.3389/fmicb.2021.596517

**Published:** 2021-02-24

**Authors:** J. Robert Logan, Kathryn M. Jacobson, Peter J. Jacobson, Sarah E. Evans

**Affiliations:** ^1^W.K. Kellogg Biological Station, Hickory Corners, MI, United States; ^2^Department of Integrative Biology, Michigan State University, East Lansing, MI, United States; ^3^Ecology, Evolution, and Behavior Program, Michigan State University, East Lansing, MI, United States; ^4^Department of Biology, Grinnell College, Grinnell, IA, United States

**Keywords:** litter decomposition, non-rainfall moisture, fog, dew, arid, dryland, carbon cycling, reciprocal transplant, climate change, Namib desert

## Abstract

Non-rainfall moisture (fog, dew, and water vapor; NRM) is an important driver of plant litter decomposition in grasslands, where it can contribute significantly to terrestrial carbon cycling. However, we still do not know whether microbial decomposers respond differently to NRM and rain, nor whether this response affects litter decomposition rates. To determine how local moisture regimes influence decomposer communities and their function, we examined fungal communities on standing grass litter at an NRM-dominated site and a rain-dominated site 75 km apart in the hyper-arid Namib Desert using a reciprocal transplant design. Dominant taxa at both sites consisted of both extremophilic and cosmopolitan species. Fungal communities differed between the two moisture regimes with environment having a considerably stronger effect on community composition than did stage of decomposition. Community composition was influenced by the availability of air-derived spores at each site and by specialization of fungi to their home environment; specifically, fungi from the cooler, moister NRM Site performed worse (measured as fungal biomass and litter mass loss) when moved to the warmer, drier rain-dominated site while Rain Site fungi performed equally well in both environments. Our results contribute to growing literature demonstrating that as climate change alters the frequency, magnitude and type of moisture events in arid ecosystems, litter decomposition rates may be altered and constrained by the composition of existing decomposer communities.

## Introduction

Decomposition of organic matter is a major source of greenhouse gas emissions in terrestrial systems and plays an essential role in ecosystem carbon and nutrient cycling (Kravchenko et al., 2017; Cavicchioli et al., 2019). Biotic decomposition rates are influenced by abiotic factors including moisture and temperature, and because bacteria and fungi are the actual agents of decomposition, their response to environmental conditions often mediates the relationship between environmental drivers and decomposition rates (Bradford et al., 2017). Since microbial decomposers differ widely both in their responses to environmental conditions and their ability to decompose plant litter (Treseder and Lennon, 2015), litter decay rates can sometimes depend on how specific decomposers respond to environmental conditions (Allison et al., 2013; Glassman et al., 2018). As climate change alters temperature and moisture regimes, litter decomposition rates and subsequent CO_2_ efflux may be controlled by the size and composition of existing decomposer communities as well as how they respond to changing abiotic conditions. Understanding when and how microbial communities are driven by different climate variables and when compositional shifts have functional consequences will help us better predict how carbon cycling will change under changing climate conditions.

One of the most important climatic factors determining microbial community structure and decomposition rates is moisture availability (Adair et al., 2008). While rainfall is a particularly important driver of litter decomposition rates (Jacobson and Jacobson, 1998; Allison et al., 2013), surface litter and soil decomposition rates are often more sensitive to the frequency and timing of precipitation than to the total amount of rainfall (Yu et al., 2019). Altered drying-rewetting cycles can stress soil microorganisms, inducing changes in both microbial physiology (Treseder and Lennon, 2015) and community structure (Evans and Wallenstein, 2012; Allison et al., 2013; Matulich et al., 2015). Community responses to drying-rewetting cycles are important in water-limited systems, such as in drylands, where organisms already face significant desiccation stress. In many arid and semi-arid systems, organisms cope with water limitation by using non-rainfall moisture (fog, dew, and atmospheric water vapor; hereafter “NRM”) as a supplemental water source (Dirks et al., 2010; Jacobson et al., 2015; Gliksman et al., 2017; Wang et al., 2017). In some grasslands, dew can occur as frequently as 95% of nights (Ritter et al., 2019) and recent work has demonstrated that NRM is an important driver of decomposition in both arid and mesic grasslands alike (Evans et al., 2020). NRM can be a particularly important moisture source for standing litter (senesced litter that has not fallen to the ground surface yet), where frequent wetting and subsequent microbial growth can “prime” litter for decomposition once it reaches the soil surface (Wang et al., 2017). Since standing litter can make up the majority of total plant litter in grasslands (Zhou et al., 2009), NRM is an important driver of carbon cycling across these systems. To date though, we do not know whether microbial communities respond differently to NRM and rain or if communities are insensitive to differences between these two moisture types. Since the frequency and intensity of rainfall and NRM are changing (Johnstone and Dawson, 2010; Niu et al., 2010; Haensler et al., 2011; Dai, 2013; Akimoto and Kusaka, 2015; Kutty et al., 2019; Hůnová et al., 2020), understanding how microbial communities respond to these different moisture sources will help us predict how arid- and semi-arid systems respond to changing climate regimes.

There are several reasons why microbial communities could differ in NRM- and rain-dominated environments. First, in many systems fog and dew occur more regularly than rainfall (Eckardt et al., 2013), so organisms that rely on NRM may not face such prolonged dry periods as those relying solely on rain, and may therefore be more sensitive to desiccation (Jacobson et al., 2015). Second, since NRM typically forms at night and in the early morning when temperatures are low, the ability to remain active at colder temperatures may be more important for organisms relying on NRM as a primary moisture source than those that are solely rainfall-dependent (Evans et al., 2020). Finally, fog can transport microbial communities that differ from those dispersing through rain and clear air (Evans et al., 2019). Distinct airborne communities in fog-dominated systems could lead to distinct communities on senesced litter compared to in non-NRM affected systems. Whether or not these factors actually lead to differences in microbial communities under NRM- versus rain-dominated conditions is currently unknown, limiting our understanding of microbial community assembly and our ability to predict how decomposition rates are responding to changing climates.

We provide the first assessment of how fungal communities are differentially influenced by NRM and rainfall. Specifically, we addressed the following research questions:

(1) How does moisture regime (NRM- versus rain-dominated) drive fungal community composition?

(2) Do changes in fungal community composition driven by moisture regime influence rates of litter decomposition?

We hypothesized that differences in the air-derived fungal communities (fungi that colonized pre-sterilized tillers) as well as selection for different moisture regimes would lead to different fungal communities under the two regimes. We further hypothesized that some fungi would specialize in a particular environment. Finally, we hypothesized that these differences in community structure would affect decomposition rates, with fungi decomposing litter faster under their native moisture regime than in non-native conditions.

## Materials and Methods

### Site Descriptions and Climatic Data Collection

**FIGURE 1:**
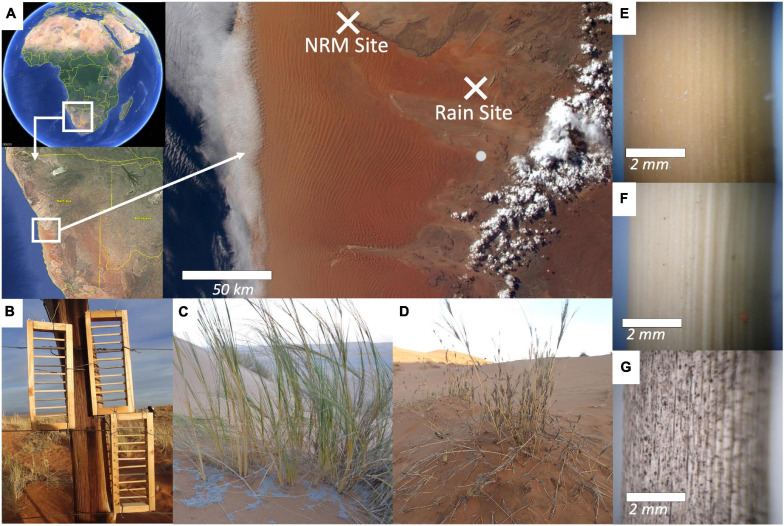
**(A)** Location of field sites in the Namib Sand Sea, with visible coastal fog in the west and rain clouds in the east (https://twitter.com/astroterry/status/ 590929048596951040), the gray dot shows the location of the Dieprivier weather station that served as a proxy weather station for the Rain Site for two months; **(B)** litter racks to minimize microclimate effects and ensure that tillers were fully exposed to sunlight and ambient moisture conditions at both locations; **(C)** living *S. sabulicola* hummock in the dunes; **(D)** standing dead *S. sabulicola* tillers will persist for years; **(E–G)** as tillers decompose, the outer cuticle is degraded and dark pigmented fungi colonize the surface.

We conducted our study in the Namib Sand Sea, a fog-affected coastal dune system in western Namibia. The Namib is ideal for studying the influence of moisture regime on microbial function because it is pristine (a sand dune system with no permanent human settlements) and has two very different moisture regimes in close proximity to one another: the western region is dominated by fog and dew and receives scant rainfall while the inland eastern areas receive more rainfall but very little non-rainfall moisture (Figure 1A). Work was conducted at the Gobabeb Research and Training Centre (NRM Site) (23°33.6′S 15°02.46′E) and at the Far East Dune (Rain Site) (23°47.04′S 15°46.86′E), approximately 75 km apart (Figure 1A). To assess differences in the abiotic environment that fungi experienced, we monitored air temperature, relative humidity, rainfall, and wetness (presence of liquid water, i.e., dew/fog) at each site. Leaf wetness sensors allowed us to determine the number of hours with liquid water on tillers. Meteorological measurements were made at each site, from weather stations situated < 50 m from where the tillers were deployed. Weather data for the NRM Site were taken from the Gobabeb Met station, part of the SASSCAL FogNet array^1^ maintained by the Gobabeb Research and Training Centre^2^. At the Rain Site, we used a HOBO data logger (H21-002, Onset Computer Corporation, United States) and sensors to record temperature and relative humidity (S-THB-M002), rain (Davis S-RGD M002), and leaf wetness (S-LWA-M003). Measurements were recorded once per minute and converted into hourly averages. The weather station at the Rain Site failed two months before the end of the experiment, so we used data from a nearby weather station (Dieprivier Namib Desert Lodge, also on the SASSCAL weather network, 40 km south). An analysis comparing weather data from the two eastern stations (Dieprivier and the Rain Site) for ten months before the failure showed that the two sites had comparable weather regimes. Since solar radiation can accelerate litter decomposition by photodegradation (King et al., 2012) and alter litter-associated decomposer communities (Pancotto et al., 2005), we estimated solar irradiance at the two sites. Since the weather stations we used did not measure solar irradiance, we used collected these data from two nearby proxy stations, both part of the SASSCAL weather network. The Rain Site’s proxy station (Dieprivier) was about 30 km SSE of the Rain Site and used a Young Model 70092 Solar Radiation sensor (R.M. Young Company, Traverse City, United States) and the NRM Site’s proxy station (Coastal Met) was 70 km NW of that site and used an NR-LITE net radiometer (Kipp and Zonen, The Netherlands). In both cases the stations are farther into their respective climatic zones, providing an upper estimate of differences in solar irradiance. Plant diversity data came from previously published work (Yeaton, 1988).

### Reciprocal Transplant Design

To see whether fungal communities functioned similarly under NRM- versus rain-dominated conditions, we reciprocally transplanted litter between the two sites (Reed and Martiny, 2007). Hereafter, we refer to “native” tillers as those deployed at the location from which they were collected (i.e., not transplanted). At the end of one year we measured mass loss, a proxy of fungal biomass, and fungal community composition of transplanted and native litter at both sites. We focused on fungi because they are considerably more abundant than bacteria on litter in this system and previous work found that fungi are important surface-litter decomposers here (Jacobson et al., 2015). We sought to identify differences in litter-associated fungal communities at the two sites, and determine what caused any differences. Specifically, we compared the importance of three potential factors that could influence litter-associated fungal communities: the species pool available as air-derived inoculum could differ between the sites, litter quality (lignin content, C:N ratios, etc.) could differ between the sites, and each site could be dominated by fungi that are particularly well suited to the meteorological conditions at that site (i.e., climatic variables could drive desiccation, thermal, and UV tolerance).

We examined native, transplanted, and autoclaved tillers of *Stipagrostis sabulicola* at the two sites. *S. sabulicola* is the dominant grass in the Namib Sand Sea and grows in hummocks on unstable dune faces (Figure 1C). Hummocks can be several meters across and persist for decades though individual tillers will senesce and fall after several years (Figure 1D). We avoided the microclimate effects associated with litter bags by deploying standing dead tillers (> 2 mm diameter) in “litter racks,” wooden frames designed to hold 9-cm long tiller pieces (mean initial mass 0.77 g ± 0.27 S.D) while exposing them to ambient moisture and solar radiation (Figure 1B). Each rack had 0.5 cm deep indentations cut into the interior so that the tillers would fit snuggly between the longest wooden pieces of the frame without having to be glued in place. Racks were covered with a dewaxed shellac to waterproof them and minimize any fungal growth on the frames themselves and placed 80–125 cm above the ground surface to mimic standing grass in a hummock. We collected tillers from various hummocks at each site covering an area of roughly 1 km^2^, within 2 km of the weather stations. Sample sizes are reported in Supplementary Table 1. After one year (June 2015–June 2016), we collected tillers, air dried them, and weighed them on an analytical balance to determine mass loss before processing them for DNA sequencing.

Despite the fact that this study was conducted in a sand dune system, sandblasting and aeolian-driven mass loss were not substantial mass loss drivers in this study. First, we used only coarse tillers, leaving no attached leafy material that would be shredded or easily removed, even during very high winds (Figure 1B). Secondly, tillers were suspended high enough above the ground (80–125 cm) that they were removed from prolonged sandblasting they would encounter if they were at the sand surface. Finally, visual observations of *S. sabulicola* tillers suspended in identical racks in other experiments we have conducted in the Namib Desert showed no signs of abrasion from sandblasting or aeolian mass loss, even after three years in the field (data not shown). We confirmed this in the present study by visually examining tillers for evidence of sand blasting after collection.

We used tillers at two different stages of decay to see whether moisture regime structured communities differently as tillers decomposed. Early-stage tillers were harvested from recently senesced stems that still had inflorescences attached (< 2 months post-senescence), were yellow, had no visible fungal growth, and had a visibly intact cuticle. Late-stage tillers were harvested from upright stems that had been standing for at least one year. These were characterized by coverings of light and dark-pigmented fungi and a cracked cuticle that was considerably more permeable to water (Jacobson et al., 2015; Figures 1E–G). The primary difference between the litter stages was that late-stage tillers had 72–444x greater fungal biomass than early-stage tillers. Since our study was confined to standing dead grass litter that had not fallen over yet, our terminology of “early” and “late” does not reflect the entire decomposition process but is meant to highlight relative successional differences between the litter types based on time since senescence and how well developed their associated fungal communities were.

To assess how colonization from air-derived fungi affected litter community assembly, we deployed “bait tillers,” which were autoclaved early-stage tillers collected from the NRM Site. We included bait tillers from only one site because space limitations in our litter racks prevented us from including bait tillers from both sites. Sterilization was confirmed by plating tillers on culture media. Autoclaving is an imperfect means of sterilization because it can alter associated chemistry but since our goal was to identify air-derived fungi, this deployment of standardized bait tillers allowed us to estimate the contribution of air-derived fungal sources (i.e., spores) since the only fungi present on autoclaved tillers after one year would have been deposited from the air. Importantly, any confounding effects of autoclaving on litter properties would have been the same for litter deployed at both sites, allowing us to use a standardized litter type to compare air-derived fungal communities at the two sites.

### Litter Physical and Chemical Analysis

We measured C:N ratios in litter on a Thermo Finnigan Flash-1112EA microanalyzer (Thermo Fisher Scientific, Waltham, MA, United States). We used an ANKOM 200 Fiber Analyzer and Daisy Incubator (ANKOM Technology, Macedon, NY, United States) to measure acid detergent lignin, cellulose, and hemicellulose of live tillers and a subset of early- and late-stage tillers to determine initial litter chemistry at the start of the study. To determine whether mass loss was driven solely by differences in leaching from tillers at the two sites, we measured leaching on a subset of recently senesced tillers collected from the two sites that were identical to those used in our decomposition study. We sealed the cut ends of each tiller with glue, submerged them in ultrapure water at 4°C for 24 h, dried them, and weighed them to quantify mass loss due to leaching.

### Molecular Methods

Fungal communities were analyzed using molecular methods. We extracted DNA from tillers using a MoBio PowerSoil DNA extraction kit supplemented with 10 min of sonication prior to mechanical lysis. We sequenced the ITS region (ITS1-F: CTTGGTCATTTAGAGGAAGTAA; ITS2: GCTGCGTTCTTCATCGATGC) using 250-bp paired-end sequencing on the MiSeq Illumina (V2) platform at Michigan State University’s Research Technology Support Facility Genomics Core. We used the same clustering and filtering pipeline used for Namib fungal (ITS) sequences described elsewhere (Evans et al., 2019). All sequences are available on NCBI’s Sequence Read Archive under BioProject number PRJNA685174.

Fungal contigs were created using default settings in fastq_mergepairs implemented in the USEARCHv8.1 pipeline^3^. Merged sequences were quality filtered to an expected error threshold of 1.0 fastq_filter (Edgar and Flyvbjerg, 2015). Sequences were then truncated to 380 bp with shorter sequences padded to reach the 380 bp because ITS region length is highly variable (Nilsson et al., 2008). Combined reads were dereplicated and OTUs were picked at the 97% identity level using UPARSE (Edgar, 2013) then chimera checked using reference based UCHIME2 (Edgar, 2016) against the UNITE 7.1 ITS1 chimera database (Kõljalg et al., 2013) within the USEARCHv9.1 pipeline.

Reads per sample ranged from 1,805 to 52,926 averaging 22,425. We used multiple extraction blanks to check for contamination, eventually removing three OTUs from the dataset. After OTU clustering, we removed any taxa that fit all three of the following criteria: it had more than 100 reads when summed across three blanks, had never been found in any previous sequencing datasets from working in the Namib Desert, and had never been cultured from the Namib in previous studies (e.g., Jacobson et al., 2015). This left us with two potential contaminants, one of which (an unknown ascomycete) was only present in a single blank but was also present in several real samples that had been processed immediately before that blank leading us to believe this was likely a crossover contaminant from a real sample so kept it in the dataset. This left only one potential contaminant that we removed from our dataset (OTU258 *Trametes versicolor* a basidiomycete found in forests). We also removed two OTUs that were identified as *S. sabulicola*, the host plant.

Since fungal reference databases have considerable errors in taxonomic identifications (Hofstetter et al., 2019), anytime we identify a specific taxon by name, we first manually verified its identity by using the NCBI’s Basic Local Alignment Search Tool, using a conservative cutoff of ≥ 97% similarity and ≥ 80% coverage (Raja et al., 2017), using only type species as references (Ko et al., 2011). Whenever possible, we verified identity by comparing our sequences of the ITS 1-2 region (250-300 bp) to those of known fungi that we had isolated from *S. sabulicola* tillers in previous studies in the Namib, which had been identified with the longer ITS 1-4 region (∼600 bp) (Jacobson et al., 2015).

As a proxy of fungal biomass, we used quantitative PCR to count the number of ITS gene copies per sample and normalized it to the tiller’s dry biomass (Song et al., 2014). While this proxy did not allow us to directly quantify fungal biomass, we were able to compare relative differences among treatment groups. For qPCR, one subset of DNA that was submitted for sequencing from each sample was diluted ten-fold and then amplified using ITS1-F/ITS2 primers and SYBR Green Supermix (BIO-RAD, United States) on the following thermocycle program: 95°C for 15 min, 40 cycles of 95°C for 30 sec then 50°C for 30 sec then 72°C for 30 s, and finally 95°C for 15 sec, 60°C for 15 sec, and ramp from 60–95° for 20 min to obtain a melting curve. We used DNA extracted from *Saccharomyces cerevisiae* as a standard to quantify ITS gene copies and normalized this to the dry mass of litter used for the extraction to produce a proxy of fungal biomass reported as log_10_ ITS copies gram^–1^.

### Statistical Analysis

We analyzed mass loss and fungal biomass data in R (R Core Team, 2019) using *t-test* and *lm* functions and analyzed the reciprocal transplant experiment with Type III ANOVAs using the *Anova* function in the *car* package. PERMANOVAs and NMDS ordinations were generated using Bray-Curtis distances calculated using the distance function in the *phyloseq* package (McMurdie and Holmes, 2013). We calculated PERMANOVAs and ordinations using relative abundance data for each taxon. We calculated Shannon-Weiner diversity and evenness using the *diversity* function in *vegan* (Oksanen et al., 2019). Correlations between taxa abundances were calculated using the *cor.test* function.

While dominance at a particular site suggests that the taxon is well-suited for that environment, high abundance could also be driven by stochastic processes or historical contingencies. To determine whether taxa that were more abundant on litter at a particular site were also those that were more common in the air at that site, we used a simple linear regression to see if the magnitude of a taxon’s greater abundance at one site over the other was correlated with its relative abundance in air-derived communities on bait tillers. For taxa with non-zero abundances at both sites, we calculated site preference as:

$$S\,i\,t\,e\,P\,r\,e\,f\,e\,r\,e\,n\,c\,e=\log_{10}\left(\frac{A_{N\,R\,M}}{A_{R\,A\,I\,N}}\right)$$

where *A*_*NRM*_ and *A*_*RAIN*_ are average relative abundances in each community based on sequence reads. A site preference value of zero means that a taxon had the same abundance at both sites while each unit above or below zero indicates a 10-fold greater relative abundance at one site. A positive site preference denotes greater abundance at the NRM Site and negative denotes greater abundance at the Rain Site.

## Results

### Site and Litter Characterization

We observed marked meteorological differences between the Rain and NRM Sites. During the one-year study, the NRM Site had 4.8× more hours of wetness and 10.7× more hours of high humidity (Table 1). Although both sites are hyperarid, the Rain Site received 5.4× more rain (56 vs. 10.3 mm yr^–1^) and more frequent rain events than the NRM Site. The average time between wet events (either rain or NRM events) was 33 hours at the NRM Site and 90 hours at the Rain Site (*p* = 0.001). Dry periods lasting for more than 100 hours were not uncommon at the Rain Site (Supplementary Figure 1). Previous work has shown that wet events are longer at the NRM Site (Evans et al., 2020); for example, at the NRM Site, only 5% of wet events lasted two hours or less while at the Rain Site 22% of wet events were this short. Mean temperature during wet events was only slightly lower at the NRM Site, which had a narrower temperature range (range 1.6–26.9°C) than did the Rain Site (range −0.7–39.1°C).

**TABLE 1:** Site differences during the one-year study period.

Value	Units	NRM Zone	Rain Zone	P
Time deployed	days	352	344	—
Rainfall	mm	10.3	56	—
Rain events ≥ 2 mm	Events	1	5	—
Rain events < 2 mm	Events	1	11	—
Rain duration	Hours	10	42	—
NRM duration	Hrs of leaf wetness	1495	311	—
Time between wet events	Hours	33	90	0.001
Time > 90% relative humidity	Hours	779	73	—
Temp when wet (range)	°C	13.0 (range 1.6–26.9)	14.8 (range -0.7–39.1)	< 0.001
Temp when dry (range)	°C	22.7 (range 3.3–42.3)	23.6 (range 0.2–42.6)	< 0.001
Mean daily temp	°C	19.7	23.2	0.008
Mean daily max	°C	29.9	32.3	< 0.001
Mean daily min	°C	13.2	14.4	0.004
Time ≥ 40°C	Hours	4	23	—
Avg Daily Solar Irradiance	MJ m^–2^	21.7	23.7	< 0.001
Perennial Grass richness	Species	1	4	
Perennial Grass Biomass (n = 14)	g m^–2^	14.9 (SE 5.8)	448.9 (SE 64.6)	

*NRM events and duration included times when leaf wetness sensors were wet but there was no recorded rain. Solar irradiance values are estimates based on data from nearby proxy locations. Plant diversity and biomass values come from Yeaton (1988). The last two months of weather data for the Rain Site came from a nearby site because of equipment failure.*

Perennial grass biomass and richness are lower at the NRM Site than at the Rain Site (Table 1). Average daily solar radiation (MJ m^–2^) during the study period was 9% greater in the vicinity of the Rain Site than the NRM Site, though these were based on estimates from proxy sites and likely overestimate differences between our sites. Both sites had similar bidirectional NW and E wind regimes with seasonal variation in wind direction, suggesting similar source regions of air-derived fungi, though the Rain Site had a stronger southern component (Supplementary Figure 2). Windspeed was greater at the NRM Site than at the Rain Site (Supplementary Figure 3), though we saw no evidence of physical damage (flaking, abrasion marks) that would indicate differences in wind damage between the two sites. Overall, the Rain Site was characterized by more frequent and longer dry periods, shorter wet periods, higher temperatures, more variable temperatures when wet, and slightly greater solar irradiance compared to the NRM Site.

Litter chemistry varied between sites and litter stages (Table 2). In live tillers, acid detergent lignin was higher and cellulose and hemicellulose concentrations were lower at the Rain Site than at the NRM Site. In both early and late senescent tillers, like those we used in the study, we did not observe significant differences in C:N, lignin:N, or percent N (Table 2). In our lab-based leaching test, early-stage tillers from the NRM Site lost more mass due to leaching than did those from the Rain Site, likely driven by increased water absorption by senescent tillers from the NRM Site (Supplementary Figure 4).

**TABLE 2:** Fungal biomass, fungal:bacterial ratios, and litter chemistry values for live *S. sabulicola* tillers and standing dead litter at both sites.

Value	Units	Live Tillers		Early-Stage Senesced		Late-Stage Senesced	
		NRM	Rain	NRM	Rain	NRM	Rain
Fungal Biomass	Log_10_(ITS copies gram^–1^)	ND	ND	4.8 ± 0.3	4.0 ± 0.2	7.1 ± 0.1	6.7 ± 0.1
Fungi:Bacteria	ITS copies : 16S copies	ND	ND	**9.5 ± 1.5**	**0.02 ± 0.01**	10.6 ± 2.4	8.2 ± 2.8
Acid Detergent Lignin	% m/m	**18.1 ± 0.6**	**23.3 ± 1.4**	21.0 ± 2.3	23.4 ± 5.0	10.6 ± 1.5	19.1 ± 6.5
Cellulose	% m/m	**44.0 ± 1.0**	**39.6 ± 0.9**	37.2 ± 2.0	33.6 ± 3.6	45.4 ± 1.1	44.1 ± 5.4
Hemicellulose	% m/m	**22.7 ± 5.3**	**19.2 ± 3.6**	**23.7 ± 0.5**	**20.3 ± 0.9**	**27.0 ± 0.5**	**20.7 ± 0.4**
Percent N	% m/m	ND	ND	0.19 ± 0.06	0.51 ± 0.23	0.29 ± 0.07	0.30 ± 0.08
C:N Ratio	Ratio m/m	ND	ND	325 ± 106	148 ± 37	191 ± 39	382 ± 254
Lignin:N Ratio	Ratio m/m	ND	ND	151 ± 53	69 ± 19	48 ± 16	331 ± 294

*Significant differences (*P* < 0.05) from *t*-tests are in bold. ND = Not Determined. Reported values are means ± S.E.M. *N* = 5 for each group.*

### Drivers of Fungal Community Composition

Fungal community richness, diversity, and evenness on native early- and late-stage litter were not significantly different between the sites, though fungal biomass was greater at the NRM Site for both litter stages (Table 3). Forty-two percent of taxa were core taxa found on native litter at both sites (Supplementary Figure 5), and these taxa make up the majority of reads (96.0% of native NRM Site reads and 88.0% of native Rain Site reads).

**TABLE 3:** Comparisons of air-derived and litter-associated fungal communities at each site. Values are only for native (i.e., non-transplanted) tillers.

Litter Type	Site	Total OTU Richness	Richness Tiller^–1^	Shannon Diversity Tiller^–1^	Evenness Tiller^–1^	Log^10^ ITS Copies g^–1^
						
Bait Tillers	NRM	177	59 ± 11.0	**2.02 ± 0.22**	**0.51 ± 0.06**	4.9 ± 0.8
	Rain	172	58.4 ± 13.5	**2.68 ± 0.16**	**0.67 ± 0.03**	3.2 ± 0.3
	*P*		0.97	**0.04**	**0.046**	0.15
Early-Stage	NRM	218	63.6 ± 5.2	1.91 ± 0.16	0.46 ± 0.04	**4.8 ± 0.3**
	Rain	159	54.6 ± 3.8	2.43 ± 0.28	0.61 ± 0.07	**4.0 ± 0.2**
	*P*		0.19	0.16	0.11	**0.04**
Late-Stage	NRM	143	53.5 ± 3.9	1.87 ± 0.19	0.47 ± 0.05	**7.1 ± 0.1**
	Rain	174	61.4 ± 12.9	1.82 ± 0.29	0.44 ± 0.05	**6.7 ± 0.1**
	*P*		0.59	0.90	0.67	**0.03**

*Values are reported as means ± 1 S.E. Bolded text denotes significant differences. Sample sizes are reported in Supplementary Table 1.*

**FIGURE 2:**
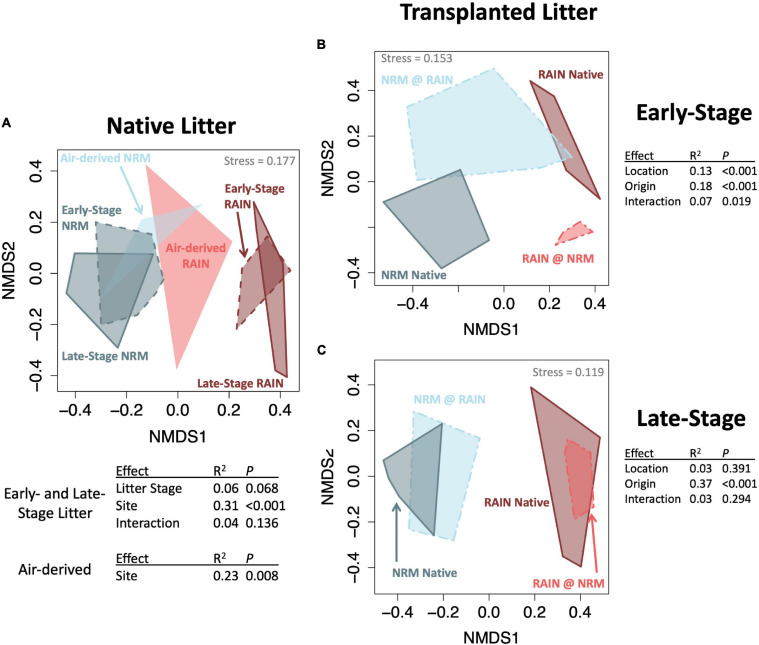
NMDS ordinations (Bray-Curtis distance) of fungal communities with groups connected by convex hulls. **(A)** Native litter and air-derived fungal communities at both sites, top table shows the PERMANOVA just for litter communities and bottom table shows results for just air-derived communities **(B)** Transplanted early-stage litter with PERMANOVA results **(C)** Transplanted late-stage litter with PERMANOVA results.

**FIGURE 3:**
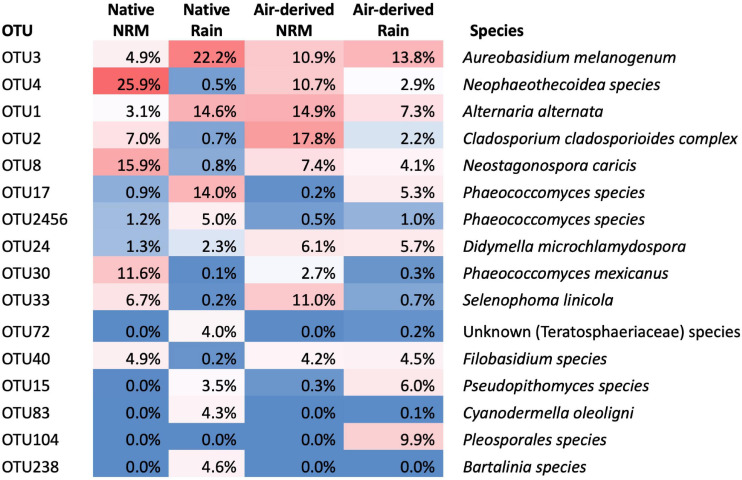
Identities of taxa that constitute the top 80% of reads across all tillers (native, transplanted, and bait tillers) and their average read abundance in native and air-derived (bait tiller) communities. Percentages for “Native” category represent the average read abundance for that OTU across both early- and late-stage tillers.

Fungal communities on native tillers differed between the sites (Figure 2A). Site explained more variation in community composition on native tillers than did litter stage (*R*^2^_*site*_ = 0.31, *R*^2^_*stage*_ = 0.06, Figure 2A). The most dominant taxa on native tillers also differed between the two sites (Figure 3). For example, at the NRM Site the three most abundant fungi on early- and late-stage litter (*Neophaeothecoidea* species (OTU4), *Neostagonospora caricis* (OTU8), and *Phaeococcomyces mexicanus* (OTU30)) together accounted for more than 53.0% of reads, but less than 1.4% at the Rain Site (Figure 3). Likewise, the top three taxa at the Rain Site (*Aureobasidium melanogenum* (OTU3), *Phaeococcomyces* sp. (OTU17), *Alternaria alternata* (OTU1)) accounted for 50.8% of Rain Site reads but only 9.2% of NRM Site reads.

**FIGURE 4:**
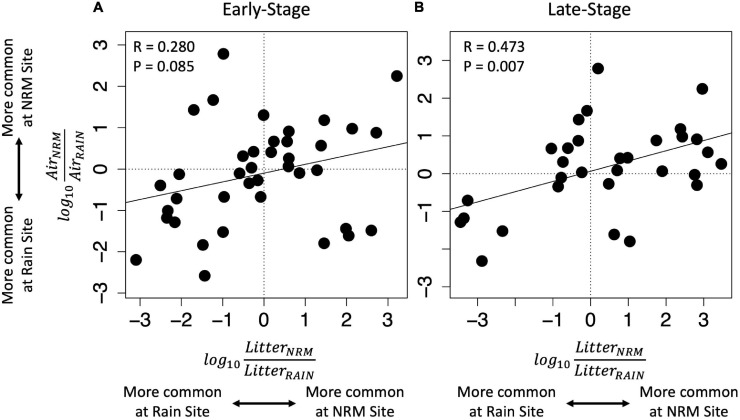
The degree of site preference on bait tillers versus site preference on native litter for taxa with non-zero abundances at both sites. Note that taxa that are abundant on native litter at one site (*x*-axis) are also more abundant on bait tillers colonized by air-derived fungi at that site (*y*-axis). This pattern is stronger for late-stage **(B)** than for early-stage tillers **(A)**. Each point represents one OTU and each unit above or below zero corresponds to a 10-fold greater relative abundance at a particular site with positive values indicating a greater relative abundance at the NRM site and negative values indicating a greater relative abundance at the Rain site. Regression lines and statistics are for simple linear regression.

Although the sites had statistically different air-derived fungal communities (*R*^2^ = 0.23, *P* = 0.008) (Figure 2A), they also shared a group of core taxa (Supplementary Figure 5); the 73 taxa that were present in the air-derived community at both sites made up 96.4% of reads at the NRM Site and 83.2% at the Rain Site. Taxa that were more common on bait tillers at each site also tended to be more common on early-stage and late-stage native litter at that site (Figure 4), suggesting that air is an important source of inoculum for tillers. Bait tillers at the NRM and Rain Sites had similar total richness, richness per tiller, and fungal biomass, though Shannon Diversity of the air-derived community was greater at the Rain Site, driven by a more even community structure (Table 3; Supplementary Figure 6).

The three most abundant air-derived taxa at the Rain Site (*Aureobasidium melanogenum* (OTU3), unknown Pleosporales (OTU104), and *Alternaria alternata* (OTU1)) made up 31.0% of the Rain Site air-derived reads and 25.8% of the NRM Site air-derived reads. The top three air-derived taxa at the NRM Site [*Cladosporium cladosporioides* complex (OTU2), *Alternaria alternata* (OTU1), and *Selenophoma linicola* (OTU33)] made up 43.7% of NRM Site air-derived reads and 10.2% of Rain Site air-derived reads (Figure 3). Some taxa, [e.g., *Alternaria alternata* (OTU1) and *Aureobasidium melanogenum* (OTU3)] were highly abundant on both bait tillers and native tillers at both sites.

**FIGURE 5:**
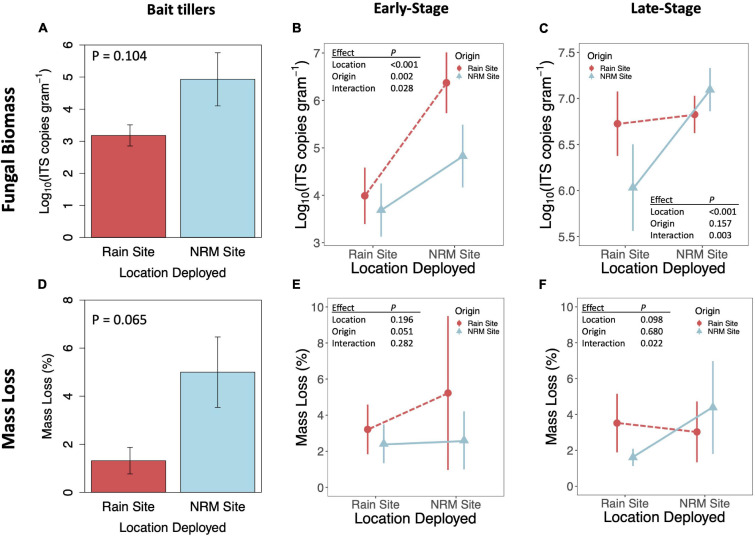
Fungal biomass as indicated by Log_10_ ITS copies gram^– 1^ (top row) and mass loss after one year (bottom row) for bait tillers **(A,D)**, early-stage tillers **(B,E)**, and late-stage tillers **(C,F)**. P-values are for t-tests for bait tillers and two-way ANOVAs for early- and late-stage tillers; full ANOVA results are reported in Supplementary Table 3. Fungal biomass statistics were run on log10 transformed data. Error bars show 95% CI. Sample sizes are included in Supplementary Table 1.

When we transplanted early-stage tillers, community composition changed to resemble native tillers at their new site, while transplanted late-stage tillers retained their native fungal communities (Figures 2B,C; PERMANOVA *P*_origin^*location_ = 0.019 for early-stage, *P*_origin^*location_ = 0.294 for late-stage). Early-stage tillers moved to the NRM Site had higher fungal biomass than those that remained at the Rain Site (Figure 5B). When late-stage tillers were moved to the Rain Site, fungal biomass was lower than for those that remained at the NRM Site (Figure 5C).

Consistent with our hypothesis that fungi were specialized to particular environments, fungi that were more common on native litter at each site increased when litter was moved to that site and decreased when litter was moved away from that site (Figure 4), though this pattern was much stronger for early-stage tillers than for late-stage tillers. We identified 38 taxa that consistently showed greater abundance at the NRM Site (i.e., they were more abundant on NRM native litter, increased when transplanted to the NRM Site, and decreased when moved away from the NRM Site) which together made up 61.2 and 77.0% of reads on early- and late-stage native litter at the NRM Site (Supplementary Table 2). Fifty-five taxa showed similar preferences for the Rain Site, making up 50.2% and 60.9% of reads on early- and late-stage native litter at the Rain Site (Supplementary Table 2).

### Functional Significance of Decomposer Communities

Fungal biomass of bait tillers tended to be higher at the NRM Site than at the Rain Site, though not significantly so (*P* = 0.104; Figure 5A) and bait tiller mass loss was 4.8x greater at the NRM Site (P = 0.065; Figure 5D). Fungal biomass at the end of the study was positively correlated with mass loss on both early- and late-stage tillers (Supplementary Figure 7). A visual inspection of tillers showed no evidence of sandblasting on the tillers upon collection. Mass loss showed a significant interaction between litter origin and deployment location for late-stage litter but not early-stage litter (Figures 5E,F). Late-stage NRM Site tillers decomposed 2.7x faster at their home (NRM) location compared to the Rain Site, but decomposition of late-stage Rain Site tillers was not significantly different between locations (Figure 5F). Early-stage litter did not decompose differently when deployed at the two sites, though we did see a marginally significant effect of origin with early-stage litter from the Rain Site moved to the NRM Site, showing greater mass loss than similarly aged litter from the NRM Site (Figure 5E). Mass loss patterns for late-stage tillers mirrored those for fungal biomass in that tillers from the Rain Site did not differ significantly whether they were transplanted or not, while NRM tillers decomposed considerably less and had significantly lower fungal biomass when they were moved to the Rain Site (Figures 5C,F).

## Discussion

Understanding how microbial communities mediate the relationship between environmental drivers and ecosystem functions can help us predict how ecosystems will respond to changing climates. Many factors influence the composition of litter-associated microbial communities including the availability of inoculum, biotic interactions among saprotrophs, the environment, and substrate quality. We have shown that, in the Namib Sand Sea, climatic differences between the NRM and Rain Sites explain differences in fungal community composition and that decomposition rates depend in part on the composition of the extant fungal community.

Throughout litter decomposition, decomposer communities typically shift in predictable ways as rapidly growing early colonizers give way to slower specialists that degrade more recalcitrant compounds (Voříšková and Baldrian, 2013; Gołȩbiewski et al., 2019). While litter moisture content influences fungal communities (Dix and Webster, 1995), in grasslands, litter age can be a more important determinant of decomposer communities than environmental drivers (Matulich et al., 2015). We found that regional climatic differences influenced community composition far more than litter stage. While litter-associated communities were only marginally different between early- and late-stage litter within each site, communities differed substantially between the sites (Figure 2A). Furthermore, tillers from the same site that differed in their time since senescence by roughly two years had highly variable litter quality but had communities that were more similar to each other than same-aged tillers at different sites. This demonstrates the importance of NRM and rain as climatic drivers of fungal communities in this system.

Fog alters the microbiology of air and factors like proximity to the ocean can affect airborne microbial community composition in coastal fog systems (Evans et al., 2019). In our study, the correlation between air-derived and litter-associated taxa abundances (Figure 4) could be driven by two factors. First, spores that are more abundant in the air may provide more inoculum for litter, making the litter communities resemble those in the air. Alternatively, abundant litter-associated fungi may produce more spores that disperse through the air, making the airborne communities resemble those on litter. Likely, both of these processes happen simultaneously, but we suspect the latter may be more important. If the former were the most important, we would expect early-stage tillers, which have more open niche space for colonization, to be more similar to air-derived communities than would late-stage tillers that are already well established. Instead, air-derived community abundances were more strongly correlated with late-stage tillers than early-stage tillers (Figure 4), suggesting that this relationship may be mainly driven by the production and release of airborne spores by abundant litter fungi across the landscape.

Two fungi stand out as abundant and widespread across all litter types at both sites. *Alternaria alternata* (OTU1) and *Aureobasidium melanogenum* (OTU3) each represented over 10% of read abundances on native or bait tillers at both sites (Figure 3) and are both globally ubiquitous species with high stress tolerances. *Alternaria alternata* is saprotrophic and pathogenic to a diverse range of agricultural crops worldwide (Adachi et al., 1993; Aradhya et al., 2001; Kgatle et al., 2019) and *Aureobasidium* is a polyextremophilic genus isolated from environments as wide ranging as hypersaline water, glacial ice, aviation fuel tanks, and in epiphytic and endophytic lifestyles worldwide (Gostinčar et al., 2014). Both of these taxa are capable of producing dark pigments, an important strategy for coping with high solar radiation and desiccation stress (Cordero and Casadevall, 2019). That these were among the most abundant taxa isolated from native and bait tillers at both sites is perhaps unsurprising given their widespread distributions and tolerance of many environmental stressors including desiccation and UV irradiation.

Other fungi, however, showed strong discrimination between the sites. Four taxa were highly abundant on native and bait tillers at the NRM Site but not at the Rain Site (Figure 3): *Cladosporium cladosporioides complex* (OTU2), *Neophaeothecoidea spp.* (OTU4), *Phaeococcomyces mexicanus* (OTU30), and *Selenophoma linicola* (OTU33). Despite their low abundance at the warmer, drier Rain Site, most of these taxa are closely related to organisms known for their desiccation and thermal tolerance. *Neophaeothecoidea* is a monotypic genus first isolated from protea plants in South Africa and, while its ecology has not yet been described in detail, it’s confamiliars in Teratosphaeriaceae occupy a wide range of habitats including highly acidic soils (Hujslová et al., 2013) and rock surfaces in Antarctic and alpine desert environments, where they grow in highly melanized forms (Ruibal et al., 2009). *Phaeococcomyces mexicanus* has been found as an epiphyte on desert shrubs (Moreno-Rico et al., 2014), a leaf endophyte (Ricks and Koide, 2019), and in Antarctic snow (de Menezes et al., 2019) and is related the black yeasts, which are notoriously tolerant of environmental stressors associated with low water activity (Gostinčar et al., 2010). *Cladosporium cladosporioides complex* is a cosmopolitan group found on decaying plant litter in aquatic habitats (Freitas, 2018), in air samples (Bensch et al., 2010), and associated with leaf litter and living leaves of dozens of plant species (Freitas, 2018). Despite its widespread distribution, *Cladosporium* may be sensitive to prolonged exposure to extreme desiccation and heat; *Cladosporium* airborne spore abundance was substantially higher in the cooler, humid winter months than during the hot, dry summer in Riyadh, Saudi Arabia (Al-Suwaine et al., 1999), though it is still present in the desert year round. *Selenophoma linicola* is a member of the Dothideales, which contain extremotolerant rock-inhabiting fungi (Ruibal et al., 2009), though most *Selenophoma spp.* have been isolated from less extreme environments such as agricultural grasses and plant litter (Vanterpool, 1947; Brokenshire and Cooke, 1975; Crous et al., 1995; Sutton and Sankaran, 1995).

Two fungi showed much greater abundances at the Rain Site than the NRM Site: *Pseudopithomyces species* (OTU15) and *Phaeococcomyces species* (OTU17). *Pseudopithomyces* is often found on dead plant litter and as a pathogen of some plants (Ariyawansa et al., 2015) including in nearby Angola (Crous and Groenewald, 2018). The closely related genus *Pithomyces* is globally distributed throughout warm climates but is particularly common in humid coastal regions (Dingley, 1962). Interestingly, the other taxon that exhibited a strong preference for the Rain Site, *Phaeococcomyces species* (OTU17), is a congeneric of a fungus with a strong preference for the NRM Site (*Phaeococcomyces mexicanus* OTU30), suggesting that the traits responsible for thriving under the NRM-dominated versus rain-dominated environment may not be so clearly delineated along taxonomic lines.

Overall, we did not find clear differences in desiccation tolerance or general stress tolerance among the fungi that preferred the NRM Site versus the Rain Site based on our literature searches. While culture-based follow-up studies may provide more information about the desiccation and thermal tolerances of these organisms, biotic interactions may also play a role in determining which taxa dominate at each site. For instance, while some NRM Site fungi may be fully capable of surviving the abiotic conditions of the warmer, drier Rain Site, they may simply be outcompeted by others with even slightly greater dessication tolerance. Similarly, Rain Site fungi appear fully capable of utilizing NRM when given the opportunity (Figures 5C,F), but over extended periods, they may be outcompeted by fungi better able to take full advantage of NRM at the low temperatures associated with that environment. Future studies examining how these fungal communities assemble over time may be able to identify how competitive dynamics and successional changes interact with climatic tolerances to structure decomposer communities.

The fact that both of these sites harbored diverse fungi associated with such wide-ranging niches was surprising, since we might have expected that only specialized fungi could tolerate the radiation and desiccation stressors of the hyperarid Namib Desert. Instead, several of the most abundant taxa were found worldwide in mesic and arid environments. This could be partially explained by considering the traits that support wide distributions. In bacteria, some of the traits that permit growth in an arid, sunny environment (such as pigmentation) correlate with large geographic ranges (Choudoir et al., 2018), since they aid in dispersal and permit individuals to grow in a wider range of environments. That many of the dominant fungi in this hyperarid landscape were globally cosmopolitan species suggests that this may be the case for some fungi as well.

By using bait tillers, instead of direct air sampling, to assess the air-derived fungal community, we focused on those fungi that were capable of colonizing senesced grass tillers. These results show that the airborne and litter-associated fungal communities appear to interact with one another, representing a shared pool of dominant fungi. Although we were unable to determine whether air-derived communities at these two sites differed primarily due to differences in local plant diversity at the two sites, long-distance dispersal from wind, or other factors, we did find that the two sites contained a large pool of shared taxa. Cosmopolitan taxa (those that were present at both sites) make up the vast majority of reads on bait tillers at both locations, suggesting that similar air-derived species pools are available to colonize litter at both sites. Overall, litter-associated communities differed between the sites much more than air-derived communities did, suggesting that post-colonization processes play a role in structuring fungal communities differently at the two sites.

Fungal communities on some transplanted tillers changed, as would be expected if some fungi performed better in one environment than the other. Fungal communities on early-stage tillers shifted while well-established late-stage tillers retained their initial communities (Figures 2B,C). That we did not see a similar shift in community composition for transplanted late-stage tillers is likely because they had such well-developed communities by the time they were moved that there was little available space for other fungi to grow. Others have shown that litter decomposition is influenced by microbial adaptation to historical moisture regimes (Allison et al., 2013; Frossard et al., 2015; Martiny et al., 2017; Glassman et al., 2018), but these studies usually only examine responses to rainfall amount. If the decrease in mass loss when late-stage NRM tillers were moved were driven solely by the reduction in water availability and not a community-specific response to the new conditions, then these tillers would have decomposed at a similar rate as native Rain Site tillers. Instead, litter decomposition rates depended on the origin of fungi (Figures 5C,F), demonstrating that the existing fungal community influences how litter decomposes under different moisture regimes.

We found that fungi responded strongly to transplantation between two sites with radically different moisture regimes. Since our study used only two sites, we cannot definitively identify moisture type (rain vs. NRM) as the sole driver of fungal community differences that we observed, though it was likely the dominant factor. We collected standing dead litter from a sand dune system and suspended it aboveground so any differences in soil type would not be a factor. NRM duration differed by a factor of five to ten between the sites while minimum and maximum temperatures were far more comparable (Table 1). The Rain Site also experienced more hours above 40°C during the study (Table 1), which could have altered communities by differentially stressing certain fungi. Although small changes in mean temperature can have outsized effects on decomposer activity (Salah and Scholes, 2011), others studies in the Namib Desert have found that NRM-driven moisture content describes the majority of variation in biological CO_2_ respiration (Jacobson et al., 2015) and litter mass loss (Evans et al., 2020), even without explicitly including temperature in their models. Furthermore, another study (Jacobson et al., 2015) found that fungi cultured from litter at the NRM Site were able to grow in the lab under a diel temperature cycle that included five hours at 50°C, substantially higher than the maximum temperatures we observed at either site during our study period (42.3°C at the NRM Site and 42.6°C at the Rain Site). Finally, disentangling the effects of temperature and moisture type is complicated by the fact that fog and dew will only form once temperatures drop below the dew point (Ritter et al., 2019), so we would expect some degree of temperature difference between the sites, at least during wet conditions. Indeed we found that while the range of temperatures while dry were very similar at the two sites, maximum temperatures when wet were considerably lower at the NRM Site (Table 1). Future studies examining fungal responses to NRM across sites could explicitly examine the role of temperature extremes to understand how fungal communities will respond in the face of both warming temperatures and altered moisture regimes.

Although we did not attempt to identify specific traits that drove differentiation in fungal communities at the two sites, our results suggest that the ability to tolerate the harsher conditions of the warmer, drier rain-dominated environment is an important driver of fungal differentiation across this gradient. The NRM-to-Rain transition may be difficult because the Rain Site is warmer, experiences less frequent wetting, greater solar irradiance, longer dry periods, and shorter NRM events (Evans et al., 2020) (Table 1 and Supplementary Figure 1), conditions that may challenge organisms requiring long wet, cool periods for metabolic activity and growth. Conversely, Rain Site fungi were able to grow and decompose litter equally effectively at both, suggesting that most litter-associated fungi in this system have the capacity to use NRM. Jacobson et al. (2015) proposed that certain physiological traits may be important for NRM-adapted fungi including cool to mesic thermal optima for growth, rapid activation in response to wetting, and efficient desiccation processes that facilitate repeated diel on-off cycling. A follow up study (Evans et al., 2020) measuring respiration from *S. sabulicola* tillers at these same locations, found that the relationship between litter gravimetric moisture content and CO_2_–C flux did not differ between the two sites, demonstrating that fungi from both sites respire similarly under NRM. Our results showing that Rain Site fungi moved to the NRM Site decompose litter at the same rate as native NRM Site fungi would seem to corroborate this. Taken as a whole, this suggests that optimization for fog and dew by NRM specialists may not reflect any particular ability to utilize NRM so much as their inability to cope with the harsher (warmer, drier) conditions associated with the Rain Site.

Microbial biomass strongly regulates decomposition at regional scales and can predict how decomposition rates respond to changing climatic conditions (Bradford et al., 2017). Fungal biomass was correlated with mass loss (Supplementary Figure 7), and the response of fungal biomass to transplantation mirrored that of mass loss (Figure 5). While fungal biomass may be a proximate control of litter decomposition rates, fungal abundance is itself ultimately controlled by how well fungi are able to tolerate local climatic conditions. Previous work has suggested that microbes that must invest more energy in stress tolerance may decompose organic matter less efficiently as they divert resources away from growth and resource acquisition (Schimel et al., 2007). Since non-rainfall moisture is a common (Ritter et al., 2019) and important driver of litter decomposition in water-limited ecosystems (Evans et al., 2020), saprotroph desiccation tolerance and specialization to use NRM may be important to terrestrial carbon cycling.

Plant litter decomposition is influenced by many factors, including photodegradation (Austin et al., 2016), litter quality (Adair et al., 2008), decomposer communities (Glassman et al., 2018), and moisture availability (Jacobson and Jacobson, 1998; Evans et al., 2020). We showed that on standing dead litter in a hyperarid landscape, the relative availability of water from NRM vs. rain structures microbial communities with important consequences for litter decomposition rates. Litter communities were affected by succession and dispersal, though most divergence in community structure was driven by specialization to specific climatic regimes. Even though most of the dominant taxa were cosmopolitan extremophiles, many fungi preferred one moisture environment over the other, suggesting that general stress tolerance traits may not fully predict how microbial communities respond to changing moisture environments. As rainfall and NRM regimes change worldwide, (Forthun et al., 2006; Johnstone and Dawson, 2010; Niu et al., 2010; Haensler et al., 2011; Dai, 2013; Akimoto and Kusaka, 2015; Kutty et al., 2019; Hůnová et al., 2020), decomposer communities may respond to climatic shifts in unique ways, altering decomposition rates differently in environments with different predominant moisture regimes.

## Data Availability Statement

The datasets presented in this study can be found in online repositories. The names of the repository/repositories and accession number(s) can be found in the article/Supplementary Material.

## Author Contributions

KJ and PJ conceived of the study, conducted fieldwork, and edited the manuscript. SE conducted data analysis and edited the manuscript. JL conducted lab work, data analysis, and wrote the manuscript. All authors contributed to the article and approved the submitted version.

## Conflict of Interest

The authors declare that the research was conducted in the absence of any commercial or financial relationships that could be construed as a potential conflict of interest.

## Footnotes

1 www.sasscalweathernet.org

2 www.gobabeb.org

3 http://drive5.com/usearch/
