# Supplementary material for: Fungal Communities on Standing Litter Are Structured by Moisture Type and Constrain Decomposition in a Hyper-Arid Grassland
Source: Front Microbiol. 2021 Feb 24;12:596517. doi: 10.3389/fmicb.2021.596517 (PMC7943874; doi:10.3389/fmicb.2021.596517)
Supplement: Supplementary file 1 [file Data_Sheet_1.docx]

Supplementary Material for:

**Fungal communities on standing litter are structured by moisture type and constrain decomposition in a hyper-arid grassland**

J. Robert Logan^1,2,3^, Kathryn M. Jacobson^4^, Peter J. Jacobson^4^, Sarah E. Evans^1,2,3^

^1^W.K. Kellogg Biological Station, 3700 East Gull Lake Drive, Hickory Corners, MI 49060, USA

^2^Department of Integrative Biology, Michigan State University, 288 Farm Lane, East Lansing, MI 48824 ,USA

^3^Ecology, Evolution, and Behavior Program, Michigan State University, 293 Farm Lane, East Lansing, MI 48824, USA

^4^Department of Biology, Grinnell College, 1116 8^th^ Avenue, Grinnell, IA 50112, USA

Corresponding author:

J. Robert Logan ([loganja3@msu.edu](mailto:loganja3@msu.edu)), 3700 East Gull Lake Drive, Hickory Corners, MI 49060, USA

**Supplemental Tables and Figures**

Table S1. Sample sizes for each treatment group in the study.

| **Litter Stage/Type** | **Origin** | **Location Deployed** | **N** |
| --- | --- | --- | --- |
| “bait” tillers | NRM Site | NRM Site | 5 |
| “bait” tillers | NRM Site | Rain Site | 5 |
| early-stage | NRM Site | NRM Site | 8 |
| early-stage | NRM Site | Rain Site | 8 |
| early-stage | Rain Site | NRM Site | 5 |
| early-stage | Rain Site | Rain Site | 5 |
| late-stage | NRM Site | NRM Site | 6 |
| late-stage | NRM Site | Rain Site | 7 |
| late-stage | Rain Site | NRM Site | 5 |
| late-stage | Rain Site | Rain Site | 5 |

Table S2. Subset of taxa that showed consistent preferences for one site by fitting three criteria: (1) higher relative abundance on native litter at that site (2) decreased in abundance when moved away from that site and (3) increased in abundance when moved to that site. This list is a subset that only includes OTUs that had an average relative abundance above 0.5% across the six groups (in total, there were 38 OTUs with consistent NRM Site preferences and 55 with consistent Rain Site preferences).

| Site Preference | OTU | Early-Stage Litter | | Late-Stage Litter | | Airborne | |
| --- | --- | --- | --- | --- | --- | --- | --- |
|  |  | NRM | RAIN | NRM | RAIN | NRM | RAIN |
| NRM | OTU4 | 23.65% | 0.98% | 28.06% | 0.02% | 10.69% | 2.90% |
| NRM | OTU33 | 10.33% | 0.36% | 3.13% | 0.01% | 10.97% | 0.72% |
| NRM | OTU40 | 8.67% | 0.45% | 1.06% | <0.01% | 4.19% | 4.48% |
| NRM | OTU8 | 6.56% | 1.64% | 25.21% | 0.01% | 7.35% | 4.06% |
| NRM | OTU2 | 5.21% | 1.30% | 8.75% | 0.01% | 17.84% | 2.19% |
| NRM | OTU88 | 2.01% | 0.00% | 1.46% | <0.01% | 1.81% | 0.01% |
| NRM | OTU166 | 0.75% | 0.10% | 4.20% | 0.00% | 1.23% | 1.53% |
| RAIN | OTU17 | 1.73% | 16.94% | 0.05% | 11.13% | 0.16% | 5.32% |
| RAIN | OTU1 | 2.13% | 6.86% | 4.13% | 22.36% | 14.86% | 7.26% |
| RAIN | OTU15 | 0.04% | 5.21% | <0.01% | 1.85% | 0.31% | 5.95% |
| RAIN | OTU2456 | 1.61% | 3.67% | 0.87% | 6.33% | 0.47% | 1.03% |
| RAIN | OTU72 | 0.01% | 1.17% | <0.01% | 6.83% | 0.04% | 0.20% |
| RAIN | OTU201 | <0.01% | 0.75% | <0.01% | 0.00% | 0.00% | 5.03% |
| RAIN | OTU382 | 0.00% | 0.58% | 0.00% | <0.01% | <0.01% | 4.40% |
| RAIN | OTU335 | <0.01% | 0.12% | 2.14% | 0.00% | <0.01% | 0.00% |
| RAIN | OTU238 | <0.01% | 0.09% | 0.00% | 9.10% | 0.00% | <0.01% |

Table S3. Type III ANOVA tables using the *Anova* function in *car* package in R. These table coincide with statistics reported on the panels in Figure 5.

Table S3_A. ANOVA table for early-stage tillers in Figure 5B

Formula: Log_10_ (ITS copies gram^-1^) ~ Location * Origin

|  | SS | Df | F | P |
| --- | --- | --- | --- | --- |
| Intercept | 548.33 | 1 | 1269 | <0.001 |
| Location | 19.08 | 1 | 44.18 | <0.001 |
| Origin | 5.23 | 1 | 12.107 | 0.002 |
| Location * Origin | 2.38 | 1 | 5.516 | 0.028 |
| Residuals | 9.50 | 22 |  |  |

Table S3_B. ANOVA table for late-stage tillers in Figure 5C

Formula: Log_10_ (ITS copies gram^-1^) ~ Location * Origin

|  | SS | Df | F | P |
| --- | --- | --- | --- | --- |
| Intercept | 1003.28 | 1 | 8545 | <0.001 |
| Location | 1.91 | 1 | 16.24 | 0.007 |
| Origin | 0.25 | 1 | 2.170 | 0.157 |
| Location * Origin | 1.31 | 1 | 11.15 | 0.003 |
| Residuals | 2.23 | 19 |  |  |

Table S3_C. ANOVA table for early-stage tillers in Figure 5E

Formula: Percent Mass Loss ~ Location * Origin

|  | SS | Df | F | P |
| --- | --- | --- | --- | --- |
| Intercept | 270.9 | 1 | 66.1 | <0.001 |
| Location | 7.307 | 1 | 1.78 | 0.196 |
| Origin | 17.56 | 1 | 4.28 | 0.051 |
| Location * Origin | 4.998 | 1 | 1.22 | 0.282 |
| Residuals | 86.1 | 21 |  |  |

Table S3_D. ANOVA table for late-stage tillers in Figure 5F

Formula: Percent Mass Loss ~ Location * Origin

|  | SS | Df | F | P |
| --- | --- | --- | --- | --- |
| Intercept | 221.8 | 1 | 90.7 | <0.001 |
| Location | 7.386 | 1 | 3.02 | 0.098 |
| Origin | 0.420 | 1 | 0.176 | 0.680 |
| Location * Origin | 15.10 | 1 | 6.177 | 0.022 |
| Residuals | 46.4 | 19 |  |  |


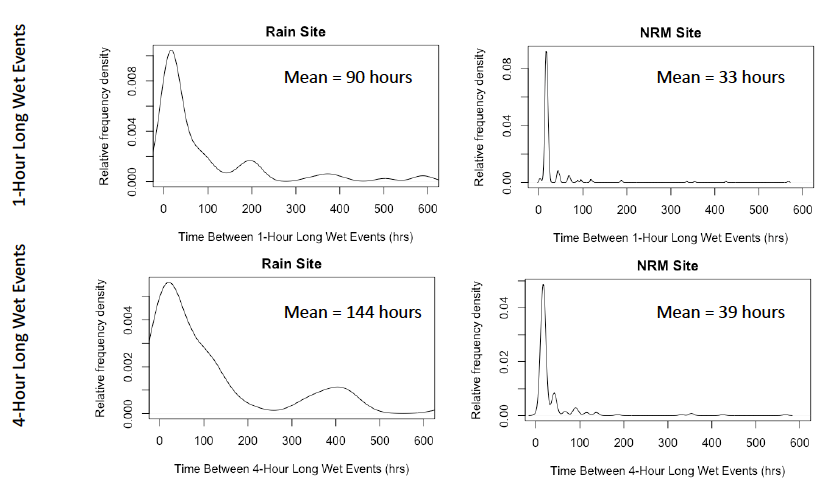


Figure S1. Frequency of dry periods between wet events at each site. Top two panels show the length of dry periods between wet events of at least one hour in length. Bottom two panels show length of dry periods between wet events of at least four hours in length.


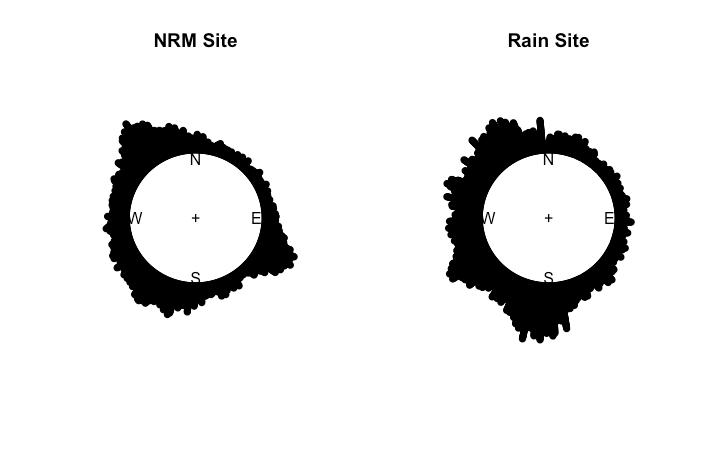


Figure S2. Wind vectors at the two sites during the study period. Length of lines on border represents the frequency (number of hours) that the wind was coming from that direction.


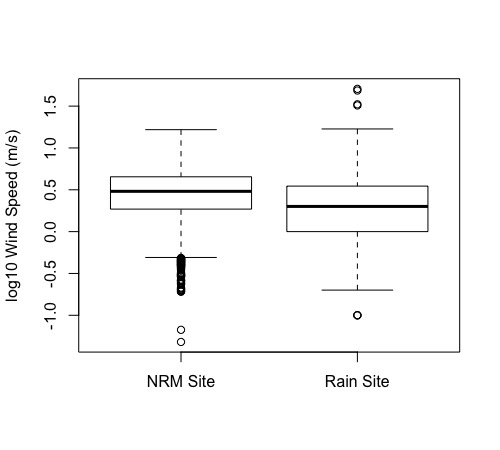


Figure S3. Log_10_ windspeed (m/s) at the two sites during the one year study based on hourly averages. Mean_NRM_ = 3.35 m/s; Mean_RAIN_ = 2.27 m/s; T = 36.1, P < 0.001. (T statistics are calculated using non-transformed data.)

Figure S4. (A) Mass loss due to leaching for a subset of early-stage tillers when submerged for 24 hours in ultrapure water after sealing the ends with glue. (B) Leachate per g of litter for the same tillers expressed as a function of gravimetric moisture. The greater mass loss from tillers at the NRM Site seen in (A) appears to be driven by greater moisture uptake (likely reflecting a more porous cuticle), not necessarily the presence of more leachable carbon.


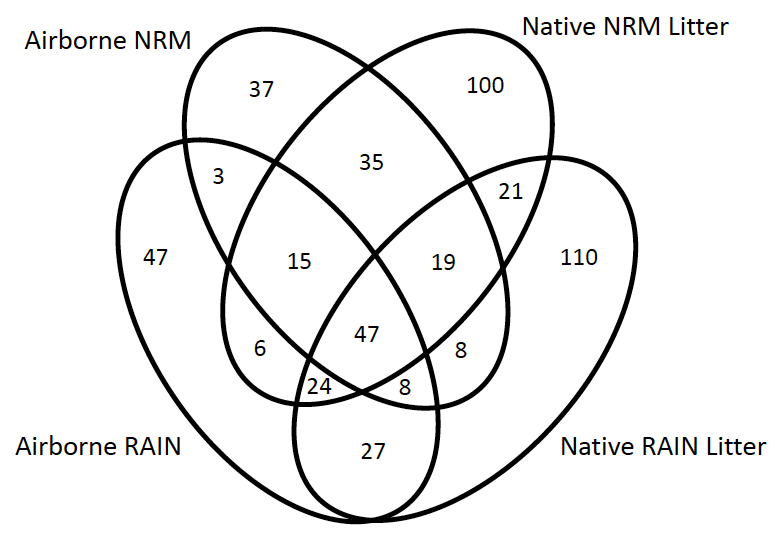


Figure S5. Venn Diagram showing overlapping fungal OTUs. For simplicity, early- and late-stage tillers are combined within each site.

Figure S6. Relative abundance distributions of taxa on tillers at each of the three litter stages. Early- and late-stage figures show only abundances on native tillers.

Figure S7. Mass loss as a function of final fungal biomass (indicated by ITS copies) for early- and late-stage senesced tillers.
